# High *ECT2* expression is an independent prognostic factor for poor overall survival and recurrence-free survival in non-small cell lung adenocarcinoma

**DOI:** 10.1371/journal.pone.0187356

**Published:** 2017-10-31

**Authors:** Shijie Zhou, Ping Wang, Xiaolan Su, Jingxia Chen, Hongfen Chen, Hanbing Yang, Aiping Fang, Linshen Xie, Yuqin Yao, Jinliang Yang

**Affiliations:** 1 Research Center for Public Health and Preventive Medicine, West China School of Public Health/No.4 West China Teaching Hospital, Sichuan University, Chengdu, China; 2 Cancer Center, West China Medical School, West China Hospital, Sichuan University, Chengdu, China; 3 Department of Emergency Medicine, the Affiliated Hospital of Weifang Medical University, Weifang, Shandong, China; University of South Alabama Mitchell Cancer Institute, UNITED STATES

## Abstract

Different subtypes of non-small cell lung cancer (NSCLC) have distinct sites of origin, histologies, genetic and epigenetic changes. In this study, we explored the mechanisms of *ECT2* dysregulation and compared its prognostic value in lung adenocarcinoma (LUAD) and lung squamous cell carcinoma (LUSC). In addition, we also investigated the enrichment of *ECT2* co-expressed genes in KEGG pathways in LUAD and LUSC. Bioinformatic analysis was performed based on data from the Cancer Genome Atlas (TCGA)-LUAD and TCGA-LUSC. Results showed that *ECT2* expression was significantly upregulated in both LUAD and LUSC compared with normal lung tissues. *ECT2* expression was considerably higher in LUSC than in LUAD. The level of *ECT2* DNA methylation was significantly lower in LUSC than in LUAD. *ECT2* mutation was observed in 5% of LUAD and in 51% of LUSC cases. Amplification was the predominant alteration. LUAD patients with *ECT2* amplification had significantly worse disease-free survival (p = 0.022). High *ECT2* expression was associated with unfavorable overall survival (OS) (*p*<0.0001) and recurrence-free survival (RFS) (*p* = 0.001) in LUAD patients. Nevertheless, these associations were not observed in patients with LUSC. The following univariate and multivariate analysis showed that the high *ECT2* expression was an independent prognostic factor for poor OS (HR: 2.039, 95%CI: 1.457–2.852, *p*<0.001) and RFS (HR: 1.715, 95%CI: 1.210–2.432, *p* = 0.002) in LUAD patients, but not in LUSC patients. Among 518 genes co-expressed with *ECT2* in LUAD and 386 genes co-expressed with *ECT2* in LUSC, there were only 98 genes in the overlapping cluster. Some of the genes related KEGG pathways in LUAD were not observed in LUSC. These differences might help to explain the different prognostic value of *ECT2* in LUAD and LUSC, which are also worthy of further studies.

## Introduction

Non-small cell lung cancer (NSCLC) is one of the leading causes of cancer death in the world [1]. NSCLC accounts for about 80% of all lung cancer cases and can be divided into three subtypes, including lung adenocarcinoma (LUAD), lung squamous cell carcinoma (LUSC) and large cell carcinoma (LCLC) [2]. Different subtypes have distinct sites of origin, histologies, genetic and epigenetic changes [3, 4]. These differences are closely related to their unique responses to therapy [5, 6]. Therefore, it is meaningful to investigate the difference in their molecular mechanisms.

Epithelial cell transforming sequence 2 (ECT2) is a guanine nucleotide exchange factor encoded by *ECT2* gene in human [7]. In non-transformed cells, ECT2 is involved in the regulation of cytokinesis via catalyzing guanine nucleotide exchange on the small GTPases, RhoA, Rac1, and Cdc42 [7]. *ECT2* is frequently upregulated in human cancers and acts as an oncogene [8, 9]. In the transformed growth of ovarian and lung cancer cells, ECT2 has distinct regulative effects from its role in cytokinesis [9–11]. Nuclear ECT2 can activate Rac1 in the cancer cells and recruit Rac effectors to the nucleus, which is required for tumor initiation and transformation [9, 11]. Knockdown of *ECT2* can inhibit Rac1 activity and block transformed growth, invasion and tumorigenicity of LUAD cells [9, 12]. One recent study found that *ECT2* upregulation was associated with worse disease-free survival and overall survival (OS) of patients with LUAD [13].

*ECT2* maps to 3q26.31 in the human genome. In fact, broad 3q chromosome amplification is the most common chromosomal aberration found in LUSC [14, 15]. In this study, via bioinformatic analysis, we explored the mechanisms of *ECT2* dysregulation in NSCLC and compared its prognostic value in LUAD and LUSC. In addition, we also investigated the enrichment of *ECT2* co-expressed genes in KEGG pathways in LUAD and LUSC.

## Materials and methods

### Bioinformatic analysis using FireBrowse

*ECT2* expression in some solid tumors and in corresponding normal tissues was analyzed by using data from the Cancer Genome Atlas (TCGA). Data analysis was performed by using FireBrowse (http://firebrowse.org/), which provides access to analyze data generated by TCGA.

### Bioinformatic analysis using UCSC Xena browser

The level 3 data of patients with primary NSCLC in TCGA-NSCLC were obtained by using the UCSC Xena browser (https://xenabrowser.net/) [16]. *ECT2* mRNA expression, exon expression and DNA methylation in patients with primary LUAD or LUSC were also examined using data in TCGA-LUAD and TCGA-LUSC, by UCSC Xena browser. Kaplan-Meier curves of OS and recurrence-free survival (RFS) after initial therapy were generated by GraphPad Prism v6.0.

### Bioinformatic analysis using cBioPortal for Cancer Genomics and ClueGo

*ECT2* genetic alteration in TCGA-LUAD and in TCGA-LUSC was examined by using cBioPortal for Cancer Genomics (http://www.cbioportal.org/) [17, 18]. The association between *ECT2* DNA mutation and disease-free survival in LUAD and LUSC patients was assessed by generating Kaplan-Meier survival curves. The genes co-expressed with *ECT2* in LUAD and LUSC (|Pearson’s r| ≥ 0.4 and |Spearman’s r| ≥ 0.4) were identified. Then, the genes were loaded into ClueGo in Cytoscape [19] for analysis of KEGG pathways. Only pathways with *p*-value ≤ 0.05 were included.

### Statistical analysis

Statistical analysis was performed by using SPSS 19.0 (SPSS Inc., Chicago, IL, USA). The association between *ECT2* RNA expression and the clinicopathological features was evaluated using χ^2^ tests. Receiver operating characteristic (ROC) curves for death and recurrence detection were constructed and the optimal cut-off value of *ECT2* expression was determined based on Youden index. Log-rank test was performed to assess the difference between the survival curves. Prognostic values were analyzed by univariate and multivariate Cox regression models. Welch’s t-test was conducted to compare *ECT2* RNA expression between LUAD and LUSC groups. *p* < 0.05 was considered to be statistically significant.

## Results

### *ECT2* was significantly upregulated in both LUAD and LUSC compared with normal lung tissues

**Fig 1:**
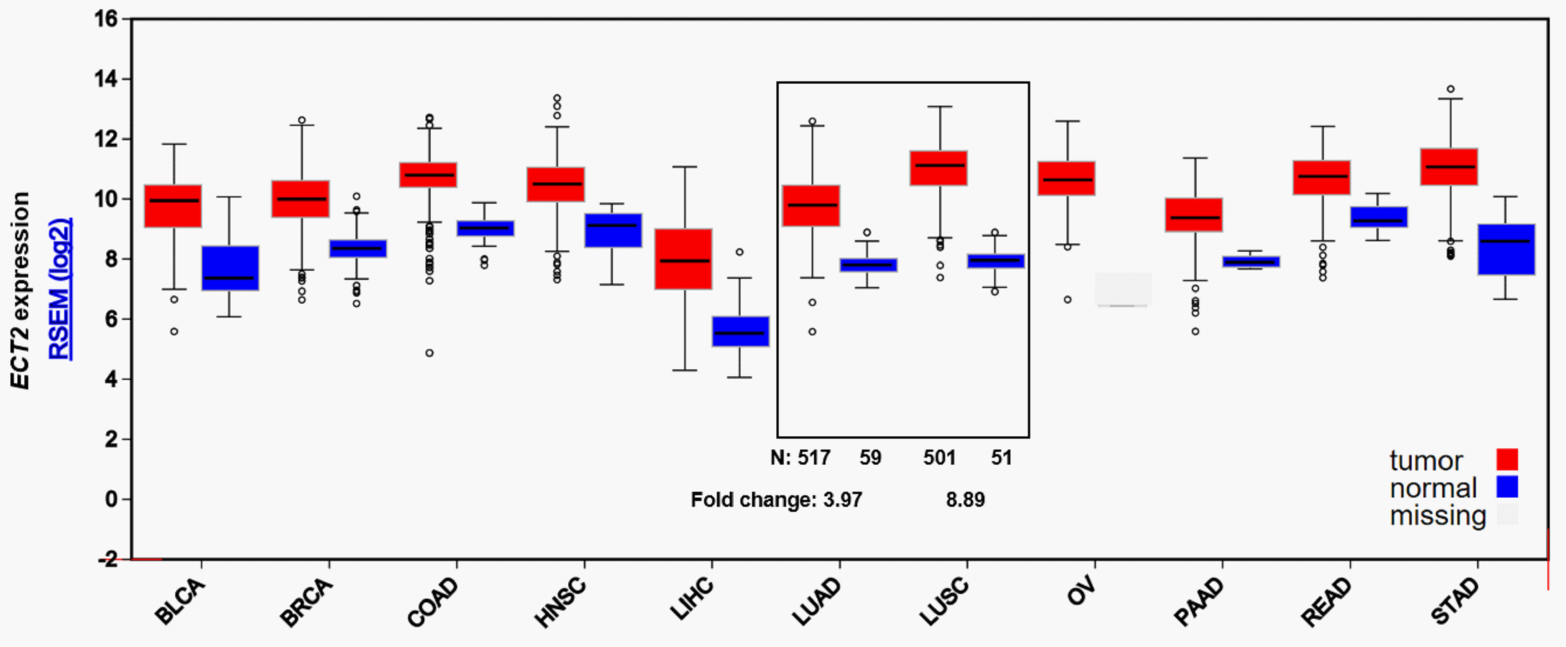
*ECT2* mRNA expression in different types of solid tumors and in corresponding normal tissues.

**Fig 2:**
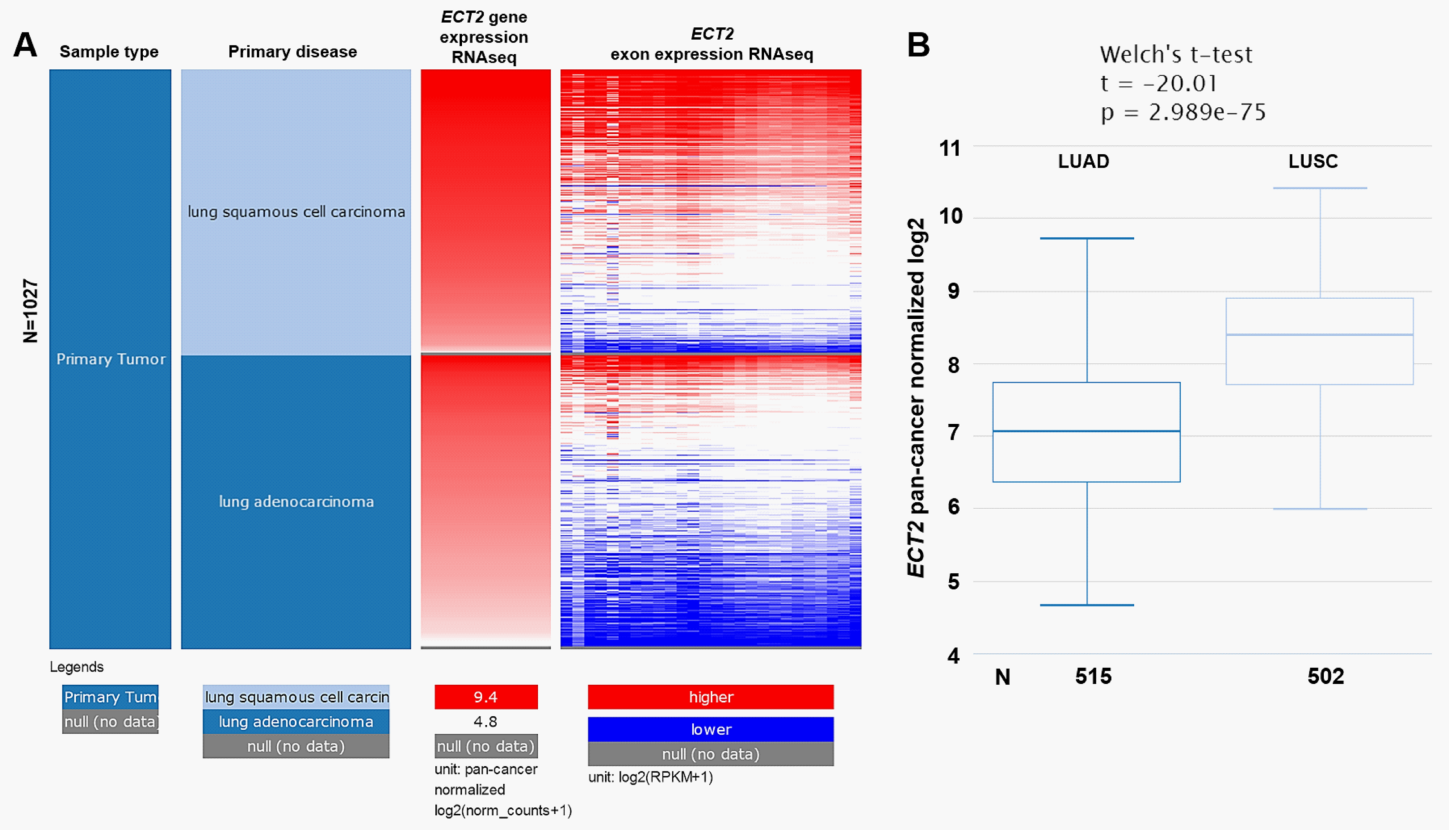
*ECT2* expression in LUSC and in LUAD. **A.** Heatmap of *ECT2* mRNA and exon expression in patients with primary LUSC or LUAD. Data were obtained from TCGA-LUSC and TCGA-LUAD. **B.** Box plots of *ECT2* expression in LUSC and in LUAD tissues. The analysis was performed using UCSC Xena Browser.

By data mining using FireBrowse, we characterized *ECT2* mRNA expression in several types of solid tumors, including LUAD and LUSC. Results indicated that *ECT2* expression was approximately 4-fold higher in LUAD tissues than in normal lung tissues, while was about 9-fold higher in LUSC tissues than in normal lung tissues (Fig 1). To further compare *ECT2* expression in LUAD and LUSC, *ECT2* mRNA RNAseq and exon RNAseq data in TCGA-LUAD and TCGA-LUSC were extracted for analysis. Heatmap and following comparison showed that *ECT2* expression was significantly higher in LUSC than in LUAD tissues (Fig 2A and 2B).

### LUSC had a lower level of *ECT2* DNA methylation and a higher level of *ECT2* DNA amplification than LUAD

**Fig 3:**
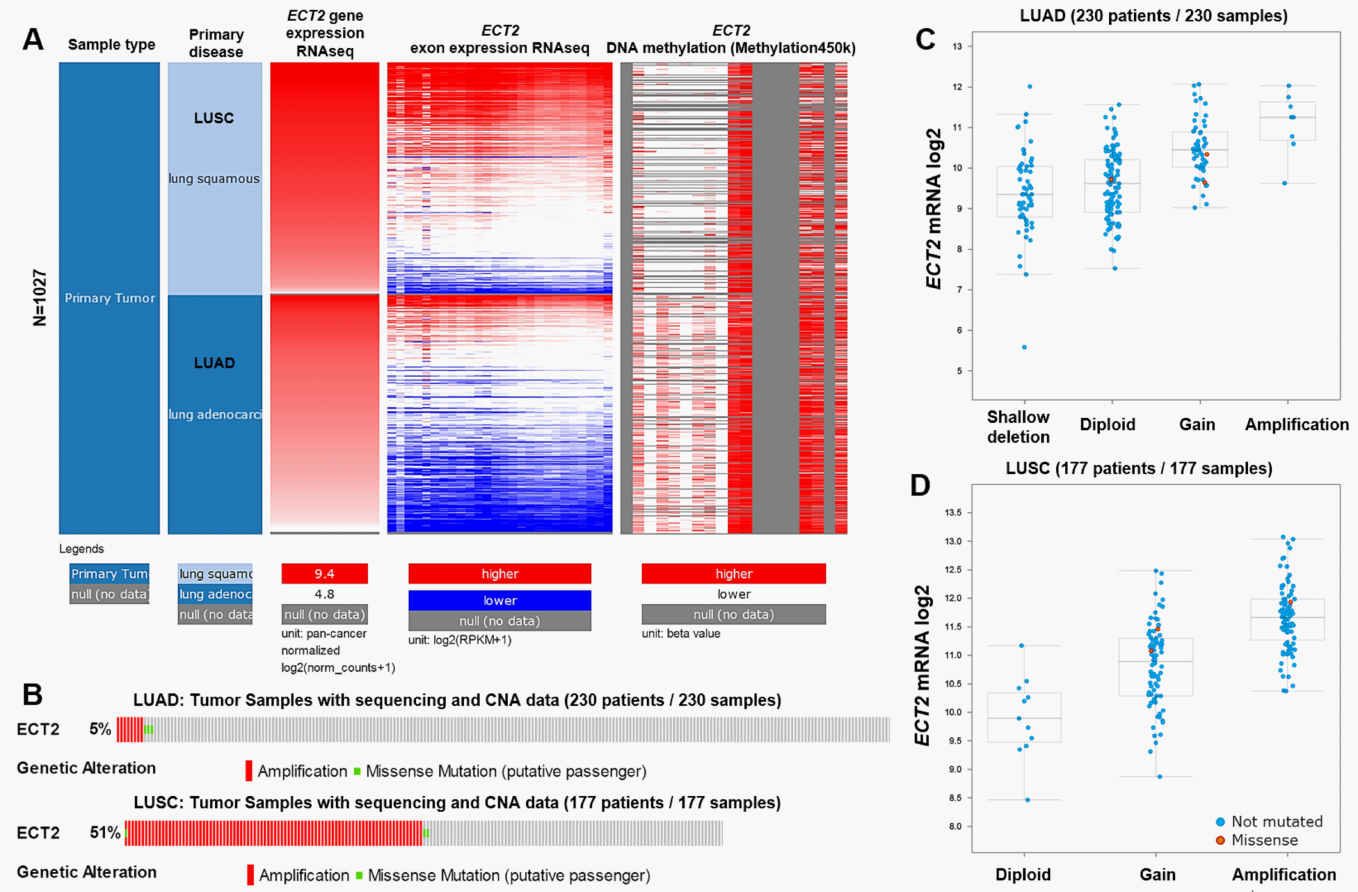
*ECT2* DNA methylation and copy number alteration (CNA) in LUSC and LUAD. **A.** Heatmap of *ECT2* mRNA expression, exon expression and DNA methylation in patients with primary LUSC or LUAD. **B.** Genetic alteration of *ECT2* in 230 cases of LUAD and 177 cases of LUSC. **C-D.** Box plots of *ECT2* expression in LUAD (C) and in LUSC (D) tissues with indicating genetic status. Data were obtained from TCGA-LUSC and TCGA-LUAD. The analysis was performed using UCSC Xena Browser and cBioPortal for Cancer Genomics.

Then, we tried to investigate the underlying mechanisms of dysregulated *ECT2* expression in LUSC and LUAD. By comparing *ECT2* expression and its DNA methylation, we observed that the level of *ECT2* DNA methylation was significantly lower in LUSC cases than in LUAD cases (Fig 3A). Some CpG loci were hypermethylated in LUAD, but not in LUSC (Fig 3A). Then, we examined copy number alterations (CNA) in TCGA-LUAD and TCGA-LUSC. *ECT2* mutation was observed in 5% of LUAD and in 51% of LUSC cases (Fig 3B). Amplification was the predominant type of alteration and was associated with increased *ECT2* mRNA expression in both LUAD and LUSC (Fig 3C and 3D).

### *ECT2* DNA mutation was associated with worse disease-free survival in LUAD, but not in LUSC patients

**Fig 4:**
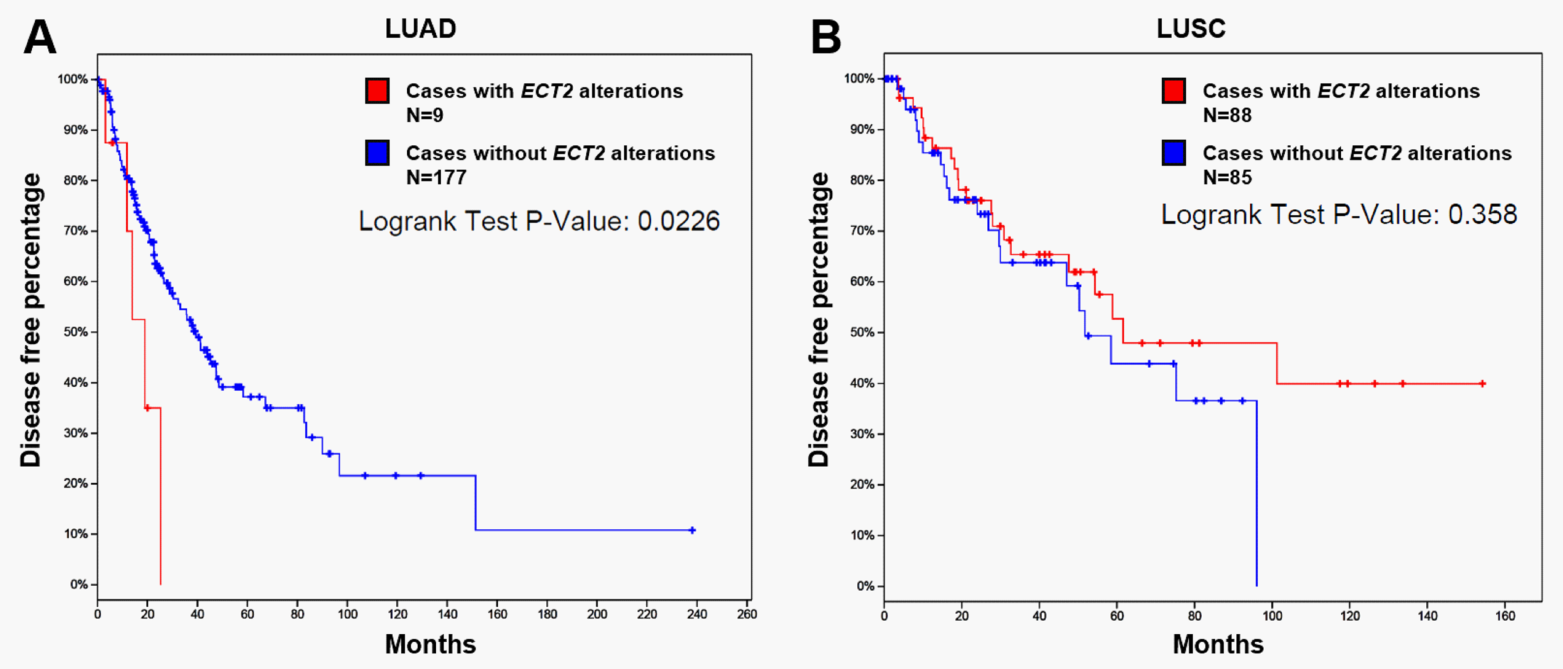
The association between *ECT2* DNA mutation and disease-free survival in LUAD (A) and LUSC (B) patients.

Then, we studied the association between *ECT2* DNA mutation and survival in LUAD and LUSC patients. Survival curves indicated that LUAD patients with *ECT2* amplification had significantly worse disease-free survival (*p* = 0.023, Fig 4A). In comparison, although *ECT2* amplification was common in LUSC patients, there was no significant association between *ECT2* amplification and disease-free survival (Fig 4B).

### High *ECT2* expression was an independent prognostic factor for poor OS and RFS in LUAD, but not in LUSC patients

**Fig 5:**
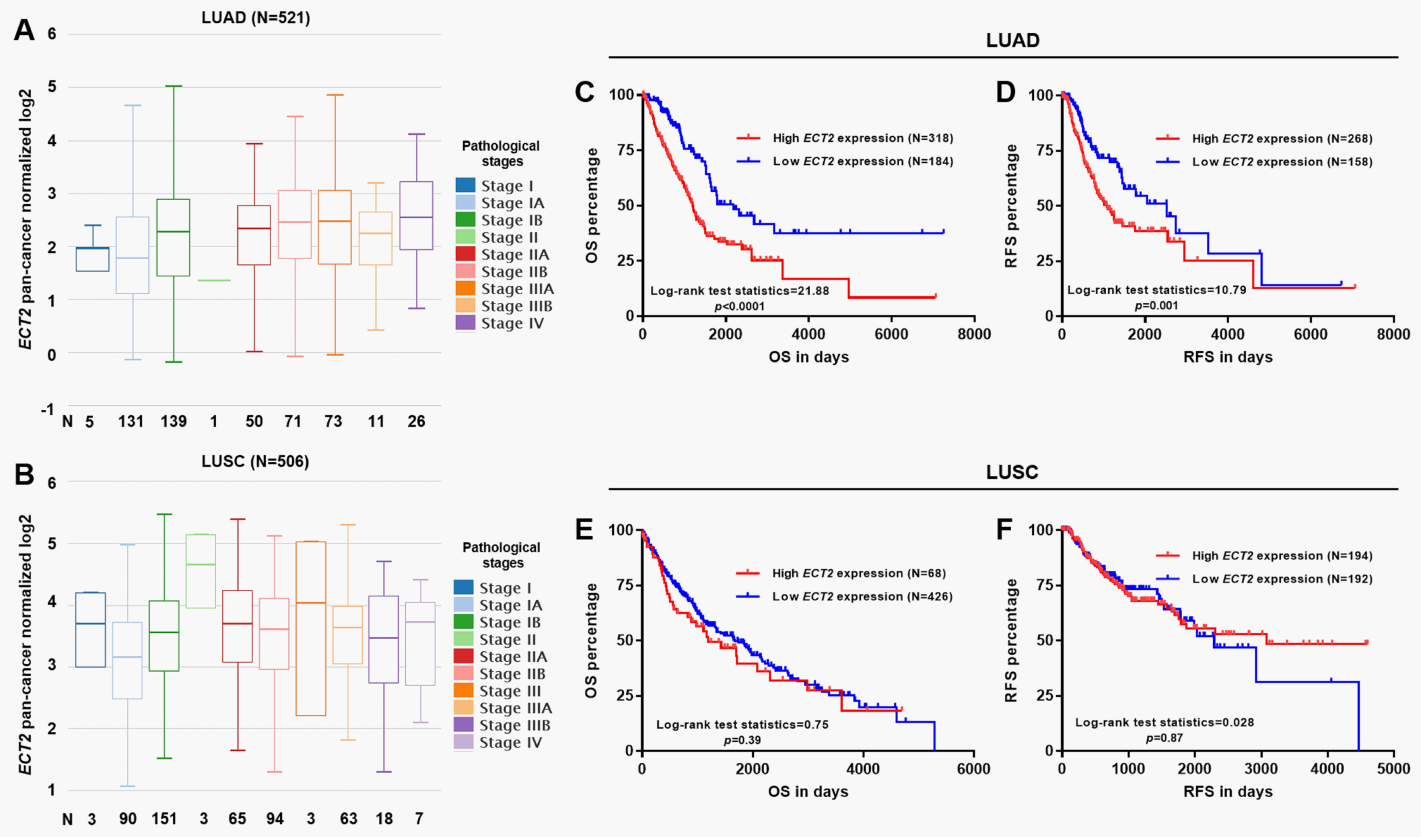
The association between *ECT2* expression and survival in LUAD and LUSC patients. **A-B.** *ECT2* expression in different pathological stages of LUAD (A) and LUSC (B). **C-F.** The association between *ECT2* expression and OS (C and E) or RFS (D and F) in LUAD (C-D) and LUSC (E-F) patients.

The associations between *ECT2* expression and the demographic and clinicopathological parameters in patients with primary LUAD and LUSC were summarized in Tables 1 and 2. In patients with LUAD, the high *ECT2* expression group had significantly lower proportions of female (156/318, 49.1% *vs*. 115/184, 62.5%, *p* = 0.0036) and lifelong non-smoker (37/311, 11.9% *vs*. 35/177, 19.8%, *p* = 0.018) than the low *ECT2* expression group (Table 1). Besides, the high *ECT2* expression group also had a significantly higher ratio of death (136/318, 42.8% *vs*. 47/184, 25.5%, *p*<0.0001) compared with the low *ECT2* expression group (Table 1). In contrast, these associations were not observed in LUSC patients (Table 2). In LUAD, *ECT2* expression gradually increased with the increase of pathological stages (Fig 5A). In comparison, this trend was not observed in LUSC (Fig 5B). High *ECT2* expression was associated with significantly worse OS (*p*<0.0001) and RFS (*p* = 0.001) in patients with LUAD (Fig 5C and 5D). Nevertheless, no significant association was observed in patients with LUSC (Fig 5E and 5F). By performing univariate analysis, we found that advanced stage (III/IV) and high *ECT2* expression were associated with significantly shorter OS and RFS in LUAD patients (Table 3). Following multivariate analysis confirmed that the high *ECT2* expression was an independent prognostic factor for poor OS (HR: 2.039, 95%CI: 1.457–2.852, *p*<0.001) and RFS (HR: 1.715, 95%CI: 1.210–2.432, *p* = 0.002) in LUAD patients (Table 3). In comparison, *ECT2* had no prognostic value in LUSC patients (Table 4).

**Table 1:** The association between *ECT2* expression and the demographic and clinicopathological parameters of patients with primary LUAD in TCGA.

Parameters		*ECT2 expression*	*ECT2 expression*	χ^2^	*p* Value
		High (N = 318)	Low (N = 184)		
Age (Mean ± SD)		64.97 ± 9.99	65.92 ± 9.89		0.31
Gender	Female	156	115	8.48	0.0036
	Male	162	69		
Smoking History	1	37	35	5.57	0.018
	2/3/4/5	274	142		
	Null	7	7		
Clinical Stage	I/II	238	150	3.18	0.075
	III/IV	75	31		
	Discrepancy+null	5	3		
Recurrence status	No	163	112	2.84	0.092
	Yes	102	49		
	Null	53	23		
Living Status	Living	182	137	14.93	<0.0001
	Dead	136	47		

**Smoking history:** 1: lifelong non-smoker; 2: current smoker; 3. Current reformed smoker (for>15 yrs); 4. Current reformed smoker (for≤15 yrs); 5. Current reformed smoker (duration not specified). Null: no data.

**Table 2:** The association between *ECT2* expression and the demographic and clinicopathological parameters of patients with primary LUSC in TCGA.

Parameters		*ECT2 expression*	*ECT2 expression*	χ^2^	*p* Value
		High (N = 68)	Low (N = 426)		
Age (Mean ± SD)		65.84 ± 8.47	67.45 ± 8.56		0.15
Gender	Female	13	115	1.9	1.38
	Male	55	311		
Smoking History	1	5	13	3.01	0.08
	2/3/4/5	62	402		
	Discrepancy+null	1	11		
Clinical Stage	I/II	56	344	0.027	0.87
	III/IV	12	78		
	Discrepancy+null	0	4		
Recurrence status	No	34	252	0.65	0.80
	Yes	15	85		
	Null	19	89		
Living Status	Living	32	250	3.24	0.07
	Dead	36	176		

**Smoking history:** 1: lifelong non-smoker; 2: current smoker; 3. Current reformed smoker (for>15 yrs); 4. Current reformed smoker (for≤15 yrs); 5. Current reformed smoker (duration not specified). Null: no data.

**Table 3:** Univariate and multivariate analyses of OS/RFS in patients with primary LUAD.

Parameters	Univariate analysis				Multivariate analysis			
	*p*	HR	95%CI (lower/upper)		*p*	HR	95%CI (lower/upper)	
**OS**								
Age > 65 *vs*.≤ 65	0.209	1.208	0.900	1.621				
Female *vs*. Male	0.670	0.939	0.702	1.256				
Smoking history 2/3/4/5 *vs*. 1	0.662	0.912	0.604	1.377				
Clinical stage III/IV *vs*. I/II	<0.001	2.646	1.942	3.606	<0.001	2.485	1.822	3.390
*ECT2* expression High *vs*. Low	<0.001	2.189	1.568	3.056	<0.001	2.039	1.457	2.852
**RFS**								
Age > 65 *vs*.≤ 65	0.081	1.340	0.964	1.863	0.028	1.455	1.042	2.030
Female *vs*. Male	0.574	1.097	0.794	1.516				
Smoking history 2/3/4/5 *vs*. 1	0.435	1.208	0.752	1.939				
Clinical stage III/IV *vs*. I/II	0.006	1.711	1.168	2.506	0.014	1.616	1.102	2.370
*ECT2* expression High *vs*. Low	0.001	1.777	1.255	2.516	0.002	1.715	1.210	2.432

**Table 4:** Univariate analysis of OS/RFS in patients with primary LUSC.

Parameters	Univariate analysis			
	*p*	HR	95%CI (lower/upper)	
**OS**				
Age > 67 *vs*.≤ 67	0.381	1.130	0.860	1.486
Female *vs*. Male	0.273	0.836	0.607	1.152
Smoking history 2/3/4/5 *vs*. 1	0.206	0.590	0.261	1.337
Clinical stage III/IV *vs*. I/II	0.006	1.564	1.135	2.155
*ECT2* expression High *vs*. Low	0.386	1.172	0.818	1.679
**RFS**				
Age > 65 *vs*.≤ 65	0.307	0.814	0.549	1.208
Female *vs*. Male	0.070	0.640	0.395	1.037
Smoking history 2/3/4/5 *vs*. 1	0.072	0.396	0.144	1.086
Clinical stage III/IV *vs*. I/II	0.004	1.999	1.241	3.218
*ECT2* expression High *vs*. Low	0.867	0.967	0.650	1.437

### *ECT2* was involved in different signaling pathways in LUAD and LUSC

**Fig 6:**
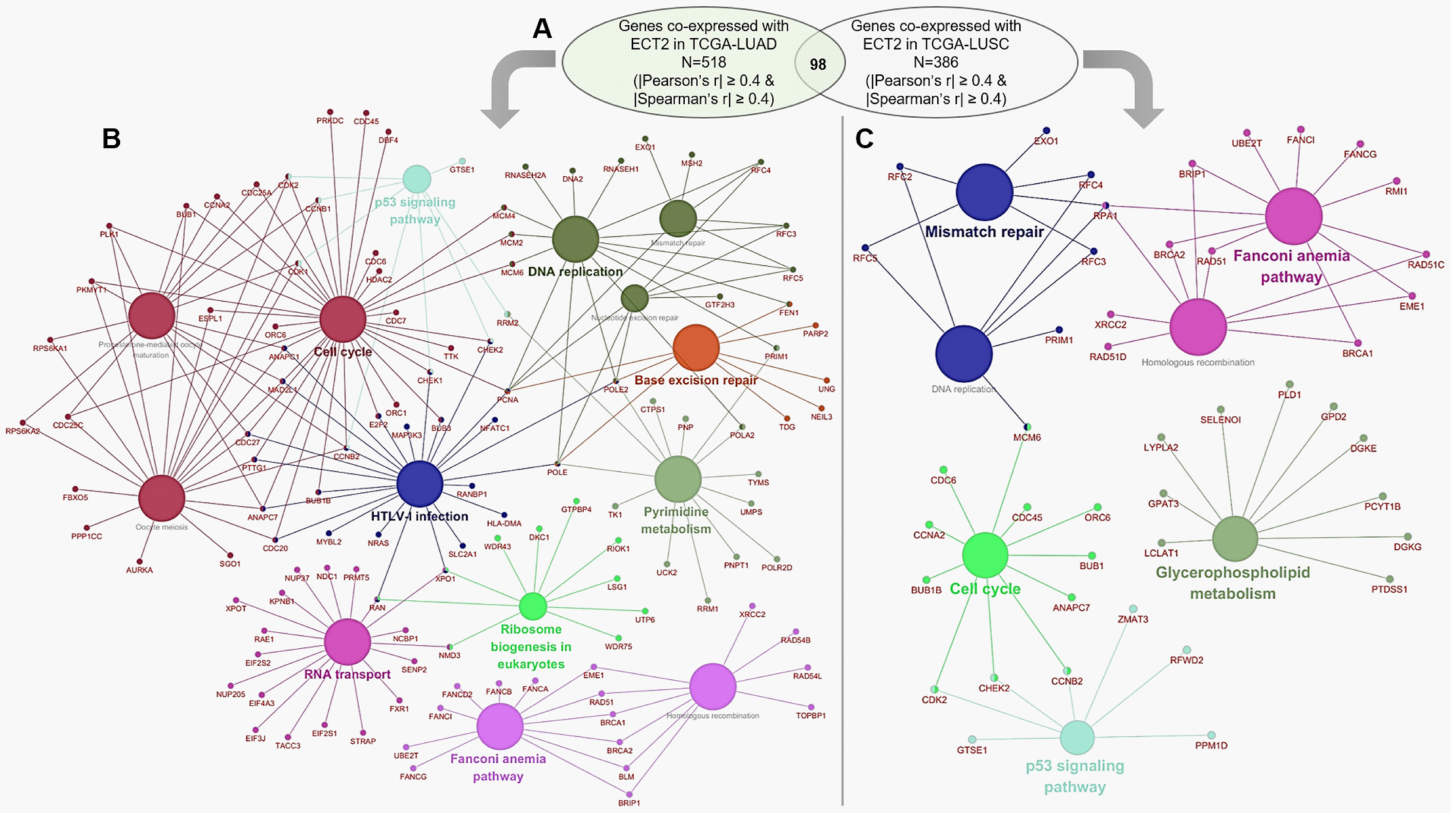
KEGG pathway analysis of the genes co-expressed with *ECT2* in LUAD and LUSC. **A.** The genes co-expressed with *ECT2* in LUAD and LUSC. **B-C.** KEGG pathway analysis of *ECT2* co-expressed genes in TCGA-LUAD (B) and in TCGA-LUSC (C).

By data mining using cBioPortal for Cancer Genomics, we identified the genes co-expressed with *ECT2* in LUAD and LUSC (|Pearson’s r| ≥ 0.4 and |Spearman’s r| ≥ 0.4) (S1 Table). Results indicated that 518 genes were co-expressed with *ECT2* in LUAD and 386 genes were co-expressed with *ECT2* in LUSC (Fig 6A and S1 Table). However, only 98 genes were in the overlapping cluster (Fig 6A). To further investigate the possible signaling pathways in which *ECT2* might be involved in, *ECT2* co-expressed genes in LUAD and LUSC were subjected to KEGG pathway analysis respectively. In LUAD, the genes were enriched in Pyrimidine metabolism, Ribosome biogenesis in eukaryotes, p53 signaling pathway, HTLV-I infection, RNA transport, Base excision repair, Homologous recombination, Fanconi anemia pathway, Cell cycle, Oocyte meiosis, Progesterone-mediated oocyte maturation, DNA replication, Nucleotide excision repair and Mismatch repair (Fig 6B and S2 Table). In comparison, *ECT2* co-expressed genes in LUSC were enriched in Glycerophospholipid metabolism, Cell cycle, p53 signaling pathway, DNA replication, Mismatch repair, Homologous recombination and Fanconi anemia pathway (Fig 6C and S3 Table).

## Discussion

Aberrant *ECT2* expression was observed in both LUAD and LUSC [13, 20, 21]. In the current study, via characterizing *ECT2* expression based on data in TCGA-LUAD and TCGA-LUSC, we also confirmed significantly upregulated *ECT2* expression in LUAD and LUSC compared with normal lung tissues. In addition, we found that *ECT2* expression was considerably higher in LUSC than in LUAD tissues. Previous studies indicated that copy number gains (CNGs) are one of the most common mechanisms of dysregulated genes at chromosome 3q26 [15, 21]. In this study, we found that amplification is common in LUSC, but not in LUAD. Approximately 50% of LUSC cases had *ECT2* amplification, but this rate was only around 5% in LUAD. These findings are consistent with the prevalence of chromosome 3q26 CNGs in LUSC and the relatively rare occurrence of 3q26 CNGs in LUAD [22]. In addition, we also observed that some CpG loci of *ECT2* gene had higher levels of methylation in LUAD than in LUSC, suggesting that epigenetic alteration is also an important mechanism of dysregulated *ECT2* in NSCLC. These results help to explain why *ECT2* expression is significantly higher in LUSC than in LUAD.

As an oncogene, *ECT2* upregulation also has prognostic values in some cancers. In patients with colorectal cancer, high expression level of *ECT2* was significantly associated with tumor size, serum CEA levels and TNM stage [23]. Kaplan-Meier survival analysis indicated that patients with high *ECT2* expression had a remarkably shorter OS [23]. High level of *ECT2* expression was also associated with poor prognosis in patients with esophageal squamous cell carcinomas [20]. One study based on patients with LUAD indicated that high *ECT2* expression was associated with unfavorable disease-free survival and overall survival [13]. However, the number of patients included in this study is relatively small (N = 88) [13]. In this study, based on large datasets in TCGA, we found that although *ECT2* amplification is common in LUSC, its mutation had no influence on survival outcomes. Nevertheless, although *ECT2* amplification was less frequent in LUAD, its mutation was associated with significantly worse disease-free survival. By generating Kaplan-Meier curves, we further demonstrated that in patients with LUAD, high *ECT2* expression was related to unfavorable OS and RFS. But no significant association was observed in patients with LUSC. In addition, our univariate and multivariate analysis showed that high *ECT2* expression was an independent prognostic factor for poor OS (HR: 2.039, 95%CI: 1.457–2.852, *p*<0.001) and RFS (HR: 1.715, 95%CI: 1.210–2.432, *p* = 0.002) in LUAD patients, but not in LUSC patients. Therefore, we hypothesized that *ECT2* might play different roles in LUAD and LUSC.

In LUAD, one recent study indicated that *ECT2* could activate rRNA synthesis by binding the nucleolar transcription factor upstream binding factor 1 (UBF1) on rDNA promoters and recruiting Rac1 and its downstream effector nucleophosmin (NPM) to rDNA [11]. However, whether other mechanisms are involved in the oncogenic properties of *ECT2* in LUAD and whether *ECT2* participates in different molecular pathways in LUAD and LUSC have not been fully revealed. By comparing *ECT2* co-expressed genes in LUAD and LUSC, we found a considerable variation. Among 518 genes co-expressed with *ECT2* in LUAD and 386 genes co-expressed with *ECT2* in LUSC, there were only 98 genes in the overlapping cluster. The following KEGG pathway analysis of the enrichment of *ECT2* co-expressed genes showed that Cell cycle, p53 signaling pathway, DNA replication, Mismatch repair, Homologous recombination and Fanconi anemia pathway are common in LUSC and LUAD. In LUAD, *ECT2* co-expressed genes were additionally enriched in some cancer-related pathways, such as Pyrimidine metabolism, Ribosome biogenesis in eukaryotes, RNA transport and Base excision repair. Therefore, it is meaningful to further investigate the involvement of *ECT2* in these pathways in LUAD in the future.

## Conclusion

Both genetic and epigenetic alterations contributed to dysregulated *ECT2* in NSCLC. High *ECT2* expression was an independent prognostic factor for poor OS and RFS in LUAD patients, but not in LUSC patients. Some of the genes related KEGG pathways in LUAD were not observed in LUSC. These differences might help to explain the different prognostic value of *ECT2* in LUAD and LUSC, which are also worthy of further studies.

## Supporting information

S1 Table The genes co-expressed with *ECT2* in LUAD and LUSC. (XLSX)

S2 Table KEGG pathway analysis of *ECT2* co-expressed genes in TCGA-LUAD. (DOCX)

S3 Table KEGG pathway analysis of *ECT2* co-expressed genes in TCGA-LUSC. (DOCX)
