# Correction: High *ECT2* expression is an independent prognostic factor for poor overall survival and recurrence-free survival in non-small cell lung adenocarcinoma

**DOI:** 10.1371/journal.pone.0196354

**Published:** 2018-04-19

**Authors:** Shijie Zhou, Ping Wang, Xiaolan Su, Jingxia Chen, Hongfen Chen, Hanbing Yang, Aiping Fang, Linshen Xie, Yuqin Yao, Jinliang Yang

There are errors in the Funding section. The grant recipient’s name should be “Yuqin Yao” instead of “Yuqing Yao.” The correct funding statement is: This work was supported by National Science Foundation of China, grant 81402564 to Yuqin Yao; National Natural Science Foundation of China, grant 81773375 to Yuqin Yao; and National High Technology Research and Development Program of China, grant 2015AA020904 to Yuqin Yao.
