# Supplementary material for: High ECT2 expression is an independent prognostic factor for poor overall survival and recurrence-free survival in non-small cell lung adenocarcinoma
Source: PLoS One. 2017 Oct 31;12(10):e0187356. doi: 10.1371/journal.pone.0187356 (PMC5663495; doi:10.1371/journal.pone.0187356)
Supplement: S2 Table — (DOCX) [file pone.0187356.s002.docx]

**S2 table. KEGG pathway analysis of *ECT2* co-expressed genes in TCGA-LUAD**

| **GOID** | **GO Term** | **Term P Value** | **% Associated Genes** | | **Nr. Genes** | | **Associated Genes Found** | |
| --- | --- | --- | --- | --- | --- | --- | --- | --- |
| GO:0000240 | Pyrimidine metabolism | 0.00 | 13.33 | 14.00 | | [CTPS1, PNP, PNPT1, POLA2, POLE, POLE2, POLR2D, PRIM1, RRM1, RRM2, TK1, TYMS, UCK2, UMPS] | |  |
| GO:0003008 | Ribosome biogenesis in eukaryotes | 0.00 | 9.43 | 10.00 | | [DKC1, GTPBP4, LSG1, NMD3, RAN, RIOK1, UTP6, WDR43, WDR75, XPO1] | |  |
| GO:0004115 | p53 signaling pathway | 0.00 | 11.59 | 8.00 | | [CCNB1, CCNB2, CDK1, CDK2, CHEK1, CHEK2, GTSE1, RRM2] | |  |
| GO:0005166 | HTLV-I infection | 0.00 | 9.38 | 24.00 | | [ANAPC1, ANAPC7, BUB1B, BUB3, CCNB2, CDC20, CDC27, CHEK1, CHEK2, E2F2, HLA-DMA, MAD2L1, MAP3K3, MYBL2, NFATC1, NRAS, PCNA, POLE, POLE2, PTTG1, RAN, RANBP1, SLC2A1, XPO1] | |  |
| GO:0003013 | RNA transport | 0.00 | 11.11 | 19.00 | | [EIF2S1, EIF2S2, EIF3J, EIF4A3, FXR1, KPNB1, NCBP1, NDC1, NMD3, NUP205, NUP37, PRMT5, RAE1, RAN, SENP2, STRAP, TACC3, XPO1, XPOT] | |  |
| GO:0003410 | Base excision repair | 0.00 | 24.24 | 8.00 | | [FEN1, NEIL3, PARP2, PCNA, POLE, POLE2, TDG, UNG] | |  |
| GO:0003440 | Homologous recombination | 0.00 | 24.39 | 10.00 | | [BLM, BRCA1, BRCA2, BRIP1, EME1, RAD51, RAD54B, RAD54L, TOPBP1, XRCC2] | |  |
| GO:0003460 | Fanconi anemia pathway | 0.00 | 21.82 | 12.00 | | [BLM, BRCA1, BRCA2, BRIP1, EME1, FANCA, FANCB, FANCD2, FANCG, FANCI, RAD51, UBE2T] | |  |
| GO:0004110 | Cell cycle | 0.00 | 28.23 | 35.00 | | [ANAPC1, ANAPC7, BUB1, BUB1B, BUB3, CCNA2, CCNB1, CCNB2, CDC20, CDC25A, CDC25C, CDC27, CDC45, CDC6, CDC7, CDK1, CDK2, CHEK1, CHEK2, DBF4, E2F2, ESPL1, HDAC2, MAD2L1, MCM2, MCM4, MCM6, ORC1, ORC6, PCNA, PKMYT1, PLK1, PRKDC, PTTG1, TTK] | |  |
| GO:0004114 | Oocyte meiosis | 0.00 | 16.94 | 21.00 | | [ANAPC1, ANAPC7, AURKA, BUB1, CCNB1, CCNB2, CDC20, CDC25C, CDC27, CDK1, CDK2, ESPL1, FBXO5, MAD2L1, PKMYT1, PLK1, PPP1CC, PTTG1, RPS6KA1, RPS6KA2, SGO1] | |  |
| GO:0004914 | Progesterone-mediated oocyte maturation | 0.00 | 16.67 | 16.00 | | [ANAPC1, ANAPC7, BUB1, CCNA2, CCNB1, CCNB2, CDC25A, CDC25C, CDC27, CDK1, CDK2, MAD2L1, PKMYT1, PLK1, RPS6KA1, RPS6KA2] | |  |
| GO:0003030 | DNA replication | 0.00 | 41.67 | 15.00 | | [DNA2, FEN1, MCM2, MCM4, MCM6, PCNA, POLA2, POLE, POLE2, PRIM1, RFC3, RFC4, RFC5, RNASEH1, RNASEH2A] | |  |
| GO:0003420 | Nucleotide excision repair | 0.00 | 14.89 | 7.00 | | [GTF2H3, PCNA, POLE, POLE2, RFC3, RFC4, RFC5] | |  |
| GO:0003430 | Mismatch repair | 0.00 | 26.09 | 6.00 | | [EXO1, MSH2, PCNA, RFC3, RFC4, RFC5] | |  |
