# Supplementary material for: High ECT2 expression is an independent prognostic factor for poor overall survival and recurrence-free survival in non-small cell lung adenocarcinoma
Source: PLoS One. 2017 Oct 31;12(10):e0187356. doi: 10.1371/journal.pone.0187356 (PMC5663495; doi:10.1371/journal.pone.0187356)
Supplement: S3 Table — (DOCX) [file pone.0187356.s003.docx]

**S3 Table. KEGG pathway analysis of *ECT2* co-expressed genes in TCGA-LUSC**

| **GOID** | **GO Term** | **Term P Value** | **% Associated Genes** | **Nr. Genes** | **Associated Genes Found** |
| --- | --- | --- | --- | --- | --- |
| GO:0000564 | Glycerophospholipid metabolism | 0.00 | 10.53 | 10.00 | [DGKE, DGKG, GPAT3, GPD2, LCLAT1, LYPLA2, PCYT1B, PLD1, PTDSS1, SELENOI] |
| GO:0004110 | Cell cycle | 0.00 | 8.87 | 11.00 | [ANAPC7, BUB1, BUB1B, CCNA2, CCNB2, CDC45, CDC6, CDK2, CHEK2, MCM6, ORC6] |
| GO:0004115 | p53 signaling pathway | 0.00 | 10.14 | 7.00 | [CCNB2, CDK2, CHEK2, GTSE1, PPM1D, RFWD2, ZMAT3] |
| GO:0003030 | DNA replication | 0.00 | 19.44 | 7.00 | [MCM6, PRIM1, RFC2, RFC3, RFC4, RFC5, RPA1] |
| GO:0003430 | Mismatch repair | 0.00 | 26.09 | 6.00 | [EXO1, RFC2, RFC3, RFC4, RFC5, RPA1] |
| GO:0003440 | Homologous recombination | 0.00 | 21.95 | 9.00 | [BRCA1, BRCA2, BRIP1, EME1, RAD51, RAD51C, RAD51D, RPA1, XRCC2] |
| GO:0003460 | Fanconi anemia pathway | 0.00 | 20.00 | 11.00 | [BRCA1, BRCA2, BRIP1, EME1, FANCG, FANCI, RAD51, RAD51C, RMI1, RPA1, UBE2T] |
